# Thermoelectric Oxide Modules (TOMs) for the Direct Conversion of Simulated Solar Radiation into Electrical Energy

**DOI:** 10.3390/ma3042801

**Published:** 2010-04-15

**Authors:** Petr Tomeš, Matthias Trottmann, Clemens Suter, Myriam Heidi Aguirre, Aldo Steinfeld, Philipp Haueter, Anke Weidenkaff

**Affiliations:** 1 Solid State Chemistry and Catalysis, EMPA, 8600 Duebendorf, Switzerland; E-Mails: matthias.trottmann@empa.ch (M.T); myriam.aguirre@empa.ch (M.H.A.); anke.weidenkaff@empa.ch (A.W.); 2 Department of Mechanical and Process Engineering, ETH Zurich, 8092 Zurich, Switzerland; E-Mail: suterc@ethz.ch (C.S.); 3 Solar Technology Laboratory, Paul Scherrer Institute, 5232 Villigen, Switzerland; E-Mail: aldo.steinfeld@ethz.ch (A.S.)

**Keywords:** thermoelectricity, thermoelectric converter, solar, heat transfer, radiation, modelling

## Abstract

The direct conversion of concentrated high temperature solar heat into electrical energy was demonstrated with a series of four–leg thermoelectric oxide modules (TOM). These temperature stable modules were not yet optimized for high efficiency conversion, but served as proof-of-principle for high temperature conversion. They were constructed by connecting two *p*- (La_1.98_Sr_0.02_CuO_4_) and two *n*-type (CaMn_0.98_Nb_0.02_O_3_) thermoelements electrically in series and thermally in parallel. The temperature gradient *ΔT* was applied by a High–Flux Solar Simulator source (HFSS) which generates a spectrum similar to solar radiation. The influence of the graphite layer coated on the hot side of the Al_2_O_3_ substrate compared to the uncoated surface on *ΔT*, *P_max_* and *η* was studied in detail. The measurements show an almost linear temperature profile along the thermoelectric legs. The maximum output power of 88.8 mW was reached for a TOM with leg length of 5 mm at ΔT = 622 K. The highest conversion efficiency *η* was found for a heat flux of 4–8 W cm^-2^ and the dependence of *η* on the leg length was investigated.

## 1. Introduction

The decrease of fossil fuel resources has motivated many research groups to seek technologies for the utilization of alternative energy sources [1,2]. Solar cells operating at 20% efficiency and covering 0.1% of the Earth’s land area would be sufficient to supply the worldwide yearly required energy [3]. The Sun as energy source can also be used by thermoelectric (TE) modules which directly convert solar heat into electricity. The advantage of TE modules compared to photovoltaic (PV) solar cells is that TE modules utilize the whole solar spectrum (IR, UV and visible radiation), while PV cells only use the UV–Vis part of the spectrum [4].

The performance of a thermoelectric material is classified by the TE Figure of Merit, *ZT = S^2^T / ρκ*, where *S* is the Seebeck coefficient, *ρ* is the electrical resistivity and *κ* is the thermal conductivity. In order to achieve a sufficient conversion efficiency *η* at the given temperature, values of at least *ZT* ~ 1 are required. The maximum conversion efficiency is thermodynamically limited by the Carnot efficiency [5]. As was shown by Yang and Caillat [5], a Figure of Merit in the range of 2 < ZT < 3 results in conversion efficiencies of ~ 50% of the Carnot efficiency. The real conversion efficiency depends not solely on the materials properties, but also on the construction and geometry of the TE device, as well as on the macroscopic heat and electronic transport.

Commercial thermoelectric devices are based on Bi_2_Te_3_ because this material exhibits a relatively high Figure of Merit [6,7]. Disadvantages of Bi_2_Te_3_ compounds are their limited chemical stability at high temperatures in air and their toxicity. Therefore, complex metal oxide ceramics as alternative materials are promising candidates for high temperature applications as they are inert at high temperatures in air, non-toxic, and low cost materials [8,9,10,11,12]. Among these oxides, Na_x_Co_2_O_4_ is especially interesting as it shows a high Figure of Merit, ZT ~ 0.8 at T = 800 K [13,14]. The production of single crystals with defined and stable stoichiometry is difficult, though. In contrast, perovskite-type materials based on manganate and cuprate can be easily synthesized with controllable composition and TE properties.

In this paper we describe the direct conversion of solar heat into electrical energy by a series of perovskite-type thermoelectric oxide modules. The influence of the leg length, the emissivity of the absorber plate, the heat flux on the maximum output power *P_max_* and the conversion efficiency *η* is investigated to assess the potential of this technology.

## 2. Experimental

*P*-type La_1.98_Sr_0.02_CuO_4_ [15] and *n*-type CaMn_0.98_Nb_0.02_O_3_ [16] materials are used to build a series of four-leg thermoelectric oxide modules with leg lengths of 4, 5 and 10 mm, respectively. The materials were prepared by a chimie douce synthesis procedure previously described [17]. The main advantage of this synthesis method, compared to the conventional solid state reaction method, is the homogeneity and purity of the product. The *p*- and *n*-type powders were characterized by X-Ray Diffraction (XRD) [15,16]. The powders were pressed into disc-shaped pellets with a diameter of 20 mm using a hydrostatic press (up to 200 kPa pressure). The *p*- and *n*-type pellets were sintered for 16 h at 1,373 K and 1,523 K, respectively.

The electrical resistivity and the Seebeck coefficient were measured with a RZ2001i Ozawa Science measurement system. The thermal conductivity was evaluated indirectly by separate measurements of the thermal diffusivity (Netzsch LFA apparatus) and the specific heat (Netzsch DSC apparatus). The electric and thermal transport properties were measured in the temperature range of 300 K < T < 800 K. A detailed description of the TE measurements is reported in [18].

**Figure 1:**
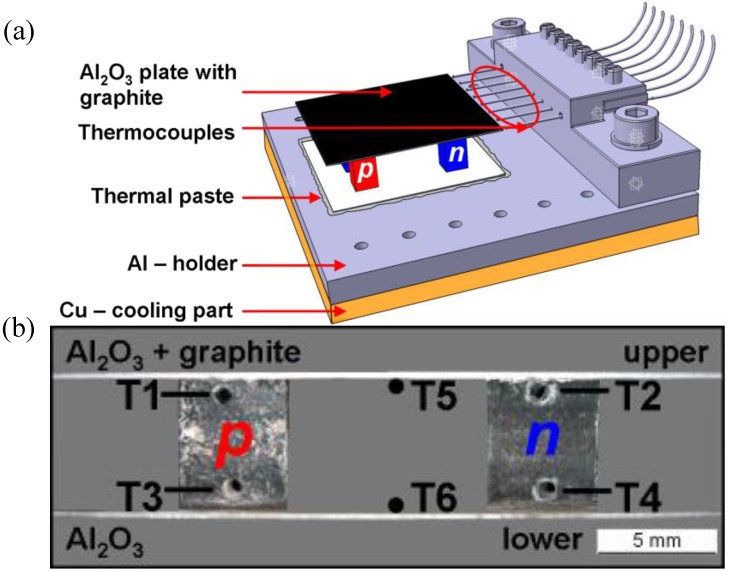
(a) Sketch of a TOM testing unit mounted on the Al-holder for the thermocouples and glued on the Cu–cooling part by means of thermal paste. The hot Al_2_O_3_ layer of the four - leg TOM is coated by a graphite layer. (b) Positions of the thermocouples in a cross-sectional profile of the 5mm TOM. 0.5 mm K—type thermocouples were placed in drilled holes.

The four–leg thermoelectric oxide modules were assembled by connecting the *p*- and *n*-type legs electrically in series and pressing them between two electrically insulated and thermally conductive Al_2_O_3_ layers [19]. A series of TOMs with different *A/l* ratio (*A* is the cross-section area and *l* is the leg length) were prepared. To Each leg had a surface area of ~ 4.5 × 4.5 mm^2^ to get an *A/l* ratio which differs from ~ 2 mm to 5 mm. For validation of the experiment two of the TOMs with the same leg length were mounted and tested. The thermal contacts were provided by two Al_2_O_3_ substrates [19].The electrical contacts between the legs and the Al_2_O_3_ layers were made by brazing with a 0.1 mm thick Ag sheet by means of a conductor paste (DuPont). The TOMs were coated on the hot side by a homogeneous black graphite layer (Figure 1a) in order to increase the absorption of solar radiation by improving the emissivity (*ε*). The 5 mm TOM 1 without coating as well as the 5 mm TOM 4 coated by SiC were measured for comparison. All the TOMs are summarized in Table 1.

**Figure 2:**
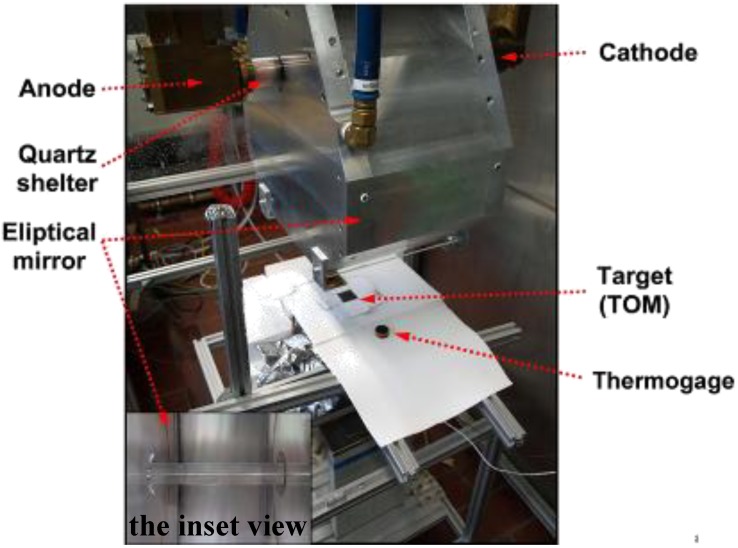
The High-Flux Solar Simulator at ETH: An elliptical mirror redirects the radiant power of the enclosed argon arc lamp onto the target (TOM).

A HFSS was used as heat source (Figure 2) [20]. A water-cooled high-pressure argon arc lamp, enclosed in a quartz tube produces radiation in the visible, infrared and ultraviolet region. The power flux intensity and the temperature can be adjusted by varying the position of the target along the axis of the focusing mirrors or by changing the electrical input power at the arc electrodes. The HFSS is able to supply a flux intensity above 500 W cm^-2^ and to provide temperatures above 3000 K. The input heat fluxes (0–14.4 W cm^-2^) were measured by a water cooled Thermogage Circular Foil Heat Flux Transducer TG1000-1 (Vatell Corporation) with a calibration range between 0–179 W cm^-2^, a sensor sensitivity of 0.084 mV W^-1^ cm^-2^ and a sensor emissivity of 0.97.

The bottom side of the TOM was cooled by cold water circulating in a Cu block. TOMs were attached to the Al–holder using a thermally conductive paste (DuPont^TM^) in order to increase the heat transfer from the cold Al_2_O_3_ layer to the Al–holder. The Al–holder itself was placed on the Cu block cooling unit with surface area of 50 × 50 mm^2^ (Figure 1a).

A series of the 0.5 mm thick K-type thermocouples were used to measure the temperature on the hot and cold side of the TOM as well as in the *p*- and *n*-type legs. One thermocouple was attached to the hot and cold Al_2_O_3_ absorber layer, respectively, and two to each TE leg by means of drilled holes with 0.6 mm in diameter (Figure 1b). The measurement accuracy of the temperature is limited by the thickness and position of the thermocouples. As the thickness of the thermocouple *d* is 0.25 mm, the measurement error of the temperature is: δT = δd*(ΔT/ Δd).

Typical measurement started by applying different heat fluxes until the temperatures at the TOM showed steady state behavior. The man value temperatures were evaluated from the time dependence of temperatures for the given heat fluxes. The voltages in the open circuit mode, the load resistances and the temperatures on the hot and cold side of the TOMs and the Al_2_O_3_ substrates were recorded simultaneously.

A test unit with a data logger was used to measure the voltage in an open circuit mode and under load resistances. The test unit consisted of four resistances connected in parallel to yield 10 loads and a digital multimeter to measure the voltages. The measurements were monitored using the software LABVIEW. The output power was calculated from the voltage and load resistance values.

## 3. Results and Discussion

**Figure 3:**
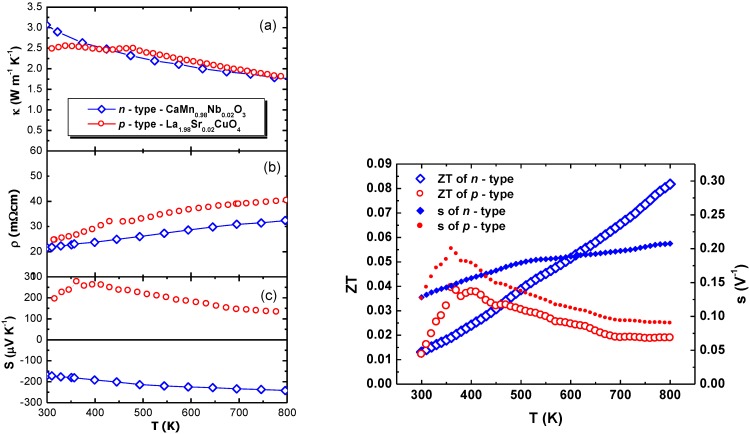
Temperature dependence of (a) the thermal conductivity, (b) the electrical resistivity, (c) the Seebeck coefficient, (d) the Figure of Merit ZT and compatibility factor s of the *p*-type (La_1.98_Sr_0.02_CuO_4_) and the *n*-type (CaMn_0.98_Nb_0.02_O_3_) TE legs.

The *p*- and *n*- type TE materials were hydrostatically pressed into pellets. They showed 90% of the theoretical density without any crack formation due to the uniformly applied pressure. A high density of the TE legs is required in order to increase the electrical conductivity and to ensure a sufficient mechanical stability of the TE modules. The thermoelectrical properties of the materials are summarized in Figure 3. The *p*- and *n*-type materials had thermal conductivities of 2.5 W m^-1^ K ^-1^ and 3 W m^-1^ K^-1^ above 300 K, respectively. Bocher *et al*. [21] measured a thermal conductivity 0.78 W m^-1^ K^-1^ lower above 300 K for the *n*-type material. The difference between the data can be attributed to modified synthesis conditions in order to obtain dense TE legs with good mechanical properties. A shorter sintering time and a lower sintering temperature (T = 1,473 K) resulted in samples with 80.77% of the theoretical density. In our case, the density exceeds 90% of the theoretical density. Further research on the relation between thermal conductivity and mechanical stability of the TE materials is needed.

Similar thermoelectric properties of the materials are a prerequisite for good conversion efficiencies of TE modules as was described previously by Snyder *et al*. [22]. The compatibility factor *s*, defined as:

(1) $$s=\frac{\left[{\left(1+ZT\right)}^{1/2}-1\right]}{ST}$$

is used to assess the similarity of the TE properties of different material. For maximum conversion efficiencies of the thermoelectric modules at large temperature gradients, the compatibility factor should not vary much with temperature [22]. Both, the *p*- and the *n*-type legs show similar metallic electrical resistivity values of *ρ_p_* = 24 mΩ cm and *ρ_n_* = 20 mΩ cm at T = 300 K, respectively. Both materials exhibit a large thermopower (*S_p_* = +200 μV K^-1^ and *S_n_* = -160 μV K^-1^) at T = 300 K.

In Figure 3d, the open symbols show the temperature dependence of the Figure of Merit *ZT* for the *p*- and *n*-type material. In the temperature range of 300 K–450 K the *p*-type material has a higher *ZT* than the *n*-type material due to its higher Seebeck coefficient. At T > 400 K the *ZT* of the *p*-type material decreases which is correlated to a decrease of the Seebeck coefficient (*S* ~ 130 μV K^-1^ at 800 K) while the *ZT* of the *n*-type material still increases due to *S* ~ -240 μV K^-1^ in the same temperature region. The closed symbols in Figure 3d show the temperature dependence of the compatibility factor *s*. The compatibility factors of both materials are perfectly matching around T = 425 K but differ by a factor of 1.3 at T = 500 K and by a factor of 2.3 at T = 800 K. This implies a decrease of the conversion efficiency of the four–leg modules at high temperature gradients.

**Figure 4:**
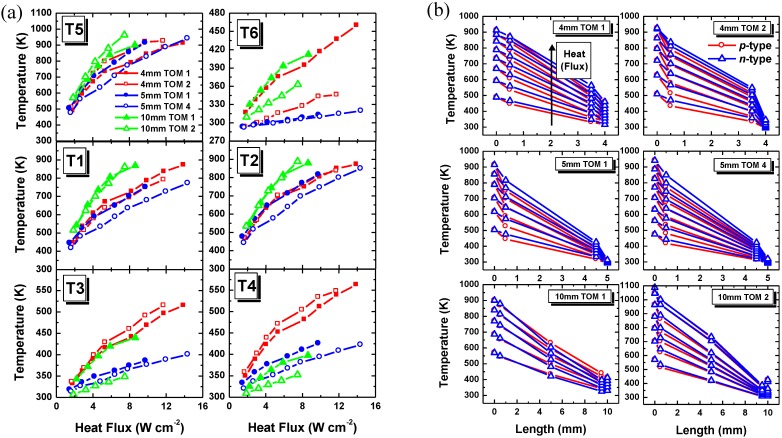
(a) Temperatures in the module as a function of the heat flux for 4, 5 and 10 mm TOMs. 5 mm TOM 4 was coated with SiC on the hot Al_2_O_3_ layer. (b) Temperature profiles along the *p*- (red circle) and *n*-type (blue triangle) legs of the 4 mm, 5 mm and 10 mm TOMs.

The average temperature along the TOMs at different measurement positions depending on the heat flux are plotted in Figure 4a for 4, 5 and 10 mm leg length. At T ~ 910 K the graphite coating on the Al_2_O_3_ absorber plate starts to decompose which results in the decrease of the temperature difference between the hot and cold side of the module as well as in the decrease of the output power and conversion efficiency (cf. Figure 6). This is caused by the lower absorptivity of the Al_2_O_3_ absorber plate when the graphite layer is declining. With increasing heat flux, the temperature on the hot side of the module (*T5*) increases as expected. All measured results are summarized in Table I.

At the same heat flux, temperatures on the hot side of the *p*- and *n*-type TE legs (*T1*, *T2*), for the same TOM, are comparable which means the manufacturing quality of the interconnections between the hot Al_2_O_3_ layer and the *p*- and *n*-type TE legs are rather reproducible and equivalent. Inducing absolute temperatures of T > 400 K, temperatures on the cold side of the module are lower in the *p*-type leg (*T3*) than the *n*-type leg (*T4*) at the same heat flux due to a higher thermal conductivity of the *p*-type material. At the cold Al_2_O_3_ plate (*T6*), temperatures are not alike which can be explained by an insufficient contact between the module and the Al-holder and deficiencies of the thermal paste. The difference is up to ~ 140 K for the 4 mm TOM 1 and the 5 mm TOM 4. The 5 mm TOM 4 was coated with SiC (*ε* ~ 0.7; total spectrum measured) [23] so the lower temperature gradient compared to the graphite-coated 5 mm TOM 1 (*ε* ~ 0.95–0.97; total spectrum measured) [23], was expectable (open blue circles in Figure 4a).

Figure 4b shows the temperature profiles along the *p*- and *n*-type legs. In the center of the 10 mm legs one additional *K*-type thermocouple was mounted (10 mm TOM 1). The error on the x-axis is estimated to be ± 0.25 mm which corresponds to an error of ~ 6% of the absolute measured temperature value. The temperature gradient along the TE legs is almost linear. The 10 mm TOM 1 is showing lower temperatures in the *n*-type leg. This abnormality can be explained by the accuracy of the temperature measurement (position and embedding of the thermocouples) and/or a slightly larger cross-section of the *p*-type legs.

**Table 1:** Summary of the properties of four–leg TOMs.

	T5_max_ [K]	T6_max_ [K]	ΔT [K]	ε [const.]	q_in, max_ [W cm^-2^]	V_OC, max_ [mV]	η [%]	P_max_ [mW]	q_in, opt_ [W cm^-2^]	η_max_ [%]
**4 mm TOM 1**	913	460	453	~ 0.95	13.90	42.9	0.043	53.7	4.06	0.065
**4 mm TOM 2**	926	346	580	~ 0.95	11.72	39.4	0.044	46.1	5.33	0.060
**5 mm TOM 1**	915	313	602	~ 0.95	9.75	44.5	0.059	51.6	7.98	0.060
5 mm TOM 4 ^*^	941	319	622	~ 0.70	14.37	46.4	0.069	88.8	7.83	0.073
**10 mm TOM 1**	901	412	489	~ 0.95	8.61	45.3	0.058	44.5	5.87	0.069
**10 mm TOM 2**	964	363	601	~ 0.95	7.46	52.4	0.081	54.4	4.26	0.082

ΔT = T5_max_–T6_max_. T5_max_ and T6_max_ are the maximum temperatures reached by applying maximum input heat flux q_in, max_; V_OC, max_, η and P_max_ are the maximum open circuit voltage, efficiency and maximum output power reached by applying maximum input heat flux q_in, max_; q_in, opt_ is the optimum input heat flux for reaching the maximum conversion efficiency η_max_. ^*^ Coated by SiC.

**Figure 5:**
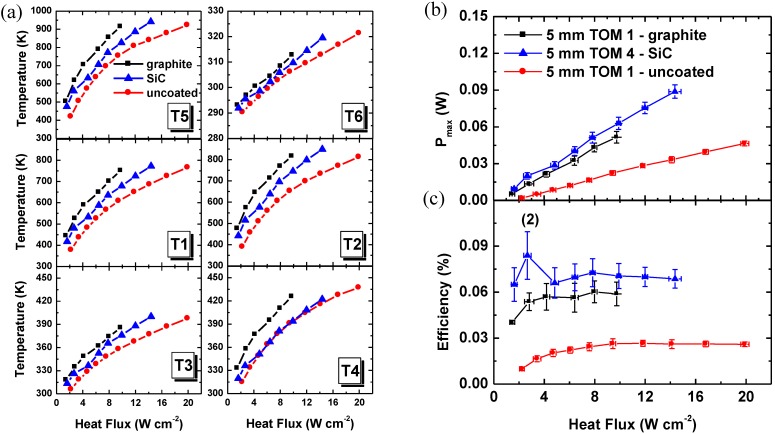
(a) Temperatures as a function of the heat flux for a graphite-coated (black squares) and an uncoated (red circles) 5 mm TOM 1, compared with a SiC-coated (blue triangles) 5 mm TOM. (b) Maximum output power and (c) conversion efficiency as function of the heat flux of a 5 mm TOM 1 and 5 mm TOM 4.

Figure 5a shows the influence of the absorber plate coating (graphite, SiC, non) on the absorptivity, the temperatures on the hot and cold Al_2_O_3_ plates, and on the temperatures in the *p*- and *n*-type TE legs using the 5 mm TOM as example. The temperature difference on the hot Al_2_O_3_ absorber plate(*T5*) between the uncoated and the graphite-coated TOM is approximately 160 K at a heat flux of 9.5 W cm^-2^. On the one hand, the heat absorption is improved by changing the emissivity of the coated surface, but on the other hand insufficient cooling on the cold side of the module leads to an increase of *T6* by 7 K at the same heat flux.

Based on the voltage measurement in open circuit mode (*V_oc_*) and at load resistances, the maximum output power (*P_max_*) was calculated considering the load resistance equal to the internal resistance [19]. *P_max_* is plotted in Figure 5b. As expected it was found that *P_max_* is higher for the graphite-coated than for the uncoated module. At the same heat flux of ~ 9.5 W cm^-2^, the temperature difference between *T5* and *T6* is 602 K and 448 K for the graphite-coated and the uncoated 5 mm TOM, respectively. Accordingly, an open circuit voltage *V_OC_* of 44.6 mV (graphite-coated) and 31.7 mV (uncoated) was measured which results in values for *P_max_* of 51.6 mW and 22.6 mW for the graphite-coated and uncoated 5 mm TOM, respectively. For the 5 mm SiC-coated TOM 4, *V_OC_* = 38.3 mV, *T5 – T6* = 507 K and *P_max_* = 60.6 mW at a heat flux of ~ 9.5 W cm^-2^ was determined. Despite the lower emissivity of SiC (ε = 0.7) compared to graphite (ε ~ 0.95) the 5 mm TOM 4 has a higher maximum output power *P_max_* even though the open circuit-voltage *V_OC_* is smaller. As the same *p*- and *n*-type materials are used for all the TOMs, this finding might be explained by small manufacturing deviations which could affect the contact resistances *R_cont_*.

Knowing the input heat flux measured by the Thermogage and the open-circuit voltage *V_oc_* measured by a test unit with a data logger, we can calculate the conversion efficiency *η*, defined by:

(2) $$\eta =\frac{V_{OC}^{2}}{4R_{load}\left(\frac{V_{OC}-V_{load}}{V_{load}}\right)Q_{in}}$$

where *V_load_* is the voltage at load resistance, *R_load_* is the load resistance, and *Q_in_* is the solar power input in [W] on the surface of the Al_2_O_3_ absorber plate. Conversion efficiencies against heat flux for the 5 mm TOMs are presented in Figure 5c. The conversion efficiencies of the graphite-coated 5 mm TOM 1 and the SiC-coated 5 mm TOM 4 are higher than the efficiency of the uncoated 5 mm TOM 1 which is in agreement with the *P_max_* results. As for the 5 mm TOM 4, the fluctuation of *η* (2^nd^ point of the blue curve in Figure 5c) was caused by a combination of the radiation fluctuation (horizontal error bars) and the instability of *V_load_* (vertical error bars).

**Figure 6:**
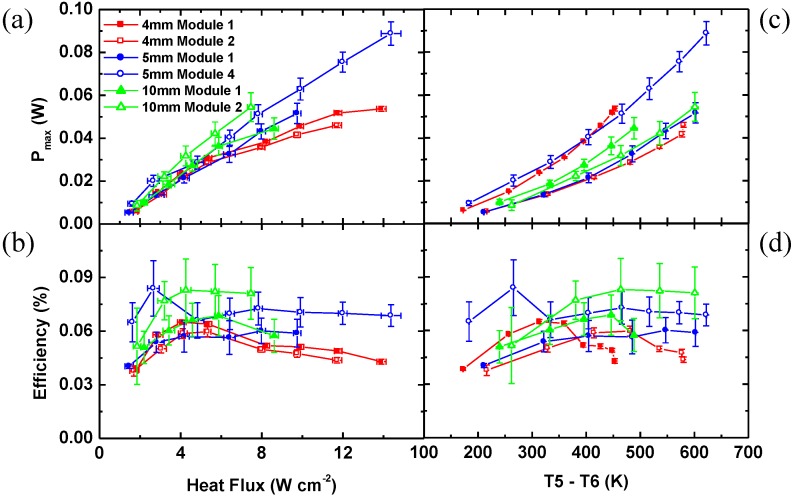
Maximum output power and conversion efficiency of the TOMs as a function of the heat flux (a, b) and the temperature difference between the hot and the cold plate (c, d).

Figure 6a-d summarize the data of *P_max_* and *η* *vs.* heat flux and *T5 – T6* for 4, 5 and 10 mm TOMs coated by graphite (5 mm TOM 4 coated by SiC). Depending on the temperature difference *T5 – T6* the maximum output power *P_max_* increases as *P_max_ ~ f* (*ΔT^2^*) and a maximum value of 54.4 mW was achieved for a leg length of 10 mm at a heat flux of 9.5 W cm^-2^ (5^th^ point in Figure 6a). A maximum output power of 88.8 mW was obtained for the 5 mm TOM 4 at a heat flux of 14.4 W cm^-2^ and a temperature difference of *T5 – T6 =* 622 K. A conversion efficiency of 0.082% was obtained for a TOM with 10 mm leg length and an Al_2_O_3_ absorber area of 30 × 30 mm^2^ (Figures 6b, d). As expected longer TE legs lead to higher conversion efficiency [24].

The conversion efficiency is higher at higher heat fluxes resulting in higher temperature differences *T5 – T6* which improves the Carnot efficiency. The conversion efficiency reaches the maximum for 4 mm TOMs around 4 W cm^-2^, for 5 mm TOMs between 4–8 W cm^-2^ and for 10 mm TOMs around 6 W cm^-2^. After reaching the maximum value, the conversion efficiency decreases because of a degradation of the graphite at high temperatures when the heat flux exceeds 7.5 W cm^-2^. A further reason for decrease of the conversion efficiency are the re-radiation losses from the Al_2_O_3_ absorber plate. The re-radiation losses from the Al_2_O_3_ absorber plate increase with *T^4^*.

As the radiation losses are difficult to measure directly, a simulation procedure was developed [25]. The results show that there is no significant radiation between the Al_2_O_3_ absorber plates and the TE legs, but the major heat losses of ~ 60% are due to the re-radiation from the hot side of the Al_2_O_3_ absorber plate.

In an ideal case the internal resistance *R_int_* of the module is equal to the resistance of the thermoelectric oxide material *R_mater_*. However, in real thermoelectric modules, the effect of the contact resistance on the conversion is not negligible. It is well known that high contact resistances lower the conversion efficiency of the devices remarkably. The internal resistances, contact resistances and resistances of the material for the TOMs were evaluated for the highest applied ΔT based on the following equations:

(3) $$R_{\text{int}}=R_{mater}+R_{cont},\;\text{where}\;R_{mater}=2\frac{l}{A}\int_{T6}^{T5}\left(<\rho_{p}>+<\rho_{n}>)(T_{5}-T_{6}\right)$$

(4) $$R_{\text{int}}=\frac{V_{OC}^{2}}{4P_{\max}}\;\text{where}\;R_{load}=R_{\text{int}}.$$

The results are summarized in Table 2 as well as the other relevant physical parameters, such as average values of Seebeck coefficient and electrical resistivity of *p*- and *n*-type TE legs for all TOMs. The internal resistance increases with leg length because of the increase of the *R_mater_*. The contact resistance vary in the range of 0.29 Ω < *R_cont_* < 0.64 Ω depending on the manufacturing quality of TOMs.

**Table 2:** Physical parameters of four–leg TOMs.

	ΔT [K]	A/l [mm]	<S_p_> [μV K^-1^]	<S_n_> [μV K^-1^]	<ρ_p_> [mΩ cm]	<ρ_n_> [mΩ cm]	R_int_ [Ω]	R_mater_ [Ω]	R_cont_ [Ω]	MQF1 [%]	MQF2 [%]
4 mm TOM 1	453	5.06	162.0	-230.8	37.8	30.0	0.86	0.27	0.59	23	31
4 mm TOM 2	580	5.06	179.3	-223.5	36.3	28.9	0.84	0.26	0.58	11	31
5 mm TOM 1	602	4.05	182.6	-220.4	35.6	28.5	0.96	0.32	0.64	14	33
5 mm TOM 4	622	4.05	179.8	-221.9	35.9	28.7	0.61	0.32	0.29	23	52
10 mm TOM 1	489	2.03	171.3	-227.4	37.1	29.4	1.15	0.66	0.49	38	57
10 mm TOM 2	601	2.03	173.2	-226.1	37.0	29.0	1.26	0.65	0.61	31	52

<S_p_>, <S_n_>, <ρ_p_> and <ρ_n_> and is the average Seebeck coefficient for *p*-type, *n*-type leg and average electrical resistivity for *p*-type, *n*-type leg, respectively.

**Figure 7:**
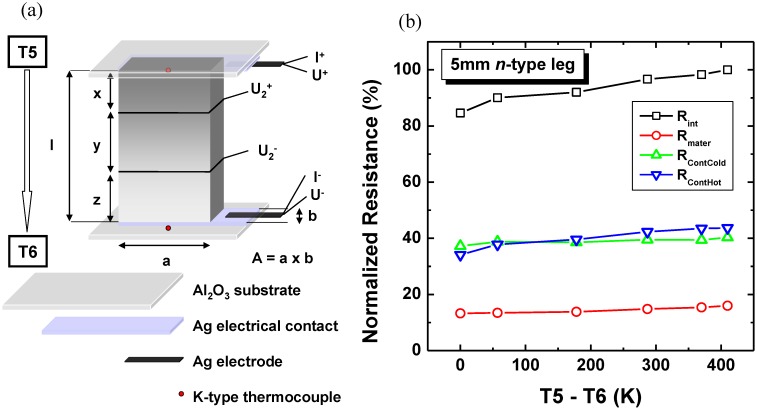
Contact resistance measurement configuration based on Equations 5-7 (a) and normalized *R_int_*, *R_mater_*, *R_contcold_* and *R_conthot_* for 5 mm *n*-type TE leg (b).

The more detailed determination of the contact resistance was done by measurement of 5 mm *n*-type TE leg at several temperature differences which vary from 0 K to 410 K. The measurement configuration is shown in Figure 7a. From the evaluation of <ρ_n_> and the internal resistance *R_int_* measurement, the contact resistance on the hot side *R_conthot_* and the cold side *R_contcold_* of the 5 mm *n*-type leg was determined. The evaluation of *R_conthot_*, *R_contcold_* and *R_int_* was done based on the following equations:

(5) $$R_{\text{int}}=\int_{T_{6}}^{T_{5}}R_{mater}\frac{d(T_{5}-T_{6})}{T_{5}-T_{6}}+R_{contcold}+R_{conthot}$$

(6) $$R_{xy}=\int_{T_{6+Z}}^{T_{5}}R_{mater,Y}\frac{d(T_{5}-T_{6+Z})}{T_{5}-T_{6+Z}}+R_{conthot}$$

(7) $$R_{yz}=\int_{T_{6+Y+Z}}^{T_{5}}R_{mater,X}\frac{d(T_{5}-T_{6+Y+Z})}{T_{5}-T_{6+Y+Z}}+R_{contcold}.$$

The data of the contact resistance measurement were normalized and plotted in Figure 7b. It was shown, that the major contribution to internal resistance is the contact resistance on the cold side and the hot side of the TOM compared to the resistance of the thermoelectric oxide materials. Thus, it can be concluded that for better conversion experiments the contacts have to be improved to decrease the contact resistance of the TOMs by e.g. developing better contact materials.

Quantitative measures of the device quality are the manufacture quality factors *MQF1*, and *MQF2* [24,26]. These factors were calculated to determine the eventual ambiguity associated with the manufacturing defects of TOMs based on the following equations:

(8) $$MQF1=\frac{P_{\text{max}}}{N\Delta T^{2}\left(S^{2}/2\rho \right)\left(A/l\right)},$$

(9) $$MQF2=\frac{R_{mater}}{R_{\text{int}}}$$

where *N* is the number of thermocouples in a TOM, *ΔT* is the temperature gradient between the hot side and the cold side of the TOM, *S* is the Seebeck coefficient, *ρ* is the electrical resistivity, *A* is the cross-sectional area of TE leg and *l* is the leg length. The manufacture quality factors vary in the range of 11% < *MQF1* < 38% and 31% < *MQF1* < 57%, respectively. The manufacture quality factor values calculated by using Equation 9 were comparable with previous studies on thermoelectric oxide modules [26]. In the case of the 5 mm TOM 1 with *P_max_* = 51.6 mW and 5 mm TOM 4 with *P_max_* = 88.8 mW it was evident that the maximum output power was enhanced by lowering the contact resistance. The manufactory quality factor measured under a similar temperature gradients of the 5 mm TOM 4 was *MQF2* = 52% while the 5 mm TOM 1 revealed a different MQF of *MQF2* = 33%.

## 4. Conclusions

Four-leg thermoelectric oxide modules, combining *p*- and *n*-type thermoelements made of La_1.98_Sr_0.02_CuO_4_ and CaMn_0.98_Nb_0.02_O_3_, respectively, were successfully used to directly convert simulated solar radiation into electrical energy by using a HFSS as energy source. The Figure of Merit ZT of the *p*- and *n*-type thermoelectric materials was evaluated up to 800 K showing nearly constant values at higher temperatures for the *p*-type and a linear increase with temperature up to ZT = 0.08 for the *n*-type material. The electrical resistivity of both materials shows metallic behavior with *ρ* between 20 - 24 mΩ cm at and |S| ≥ 160 μV K^-1^ at T = 300 K. The temperature gradient along the TE legs was almost linear showing a lower value for the *p*-type legs compared to the *n*-type legs due to a higher thermal conductivity of the *p*-type material above T = 400 K.

It was shown that coatings of the hot Al_2_O_3_ absorber plate by graphite induced a larger temperature gradient in the TOMs and the maximum output power and the conversion efficiency were significantly improved. A heat flux between 4–8 W cm^-2^ resulted in the highest conversion efficiency. The maximum conversion efficiency of 0.082% was obtained for a TOM with 10 mm leg length. With a slightly modified geometry of the TOM the conversion efficiency would be ~ 0.4% [25]. It was found that the contact resistances which vary in the range of 0.29 Ω < *R_cont_* < 0.64 Ω are limiting the conversion efficiency significantly. Thus, besides the necessity of the development of better thermoelectric materials and the lowering of re-radiation losses, a major part in the improvement of solar thermoelectric converters applied at high temperatures with concentrated solar radiation will be to reduce substantially the contact resistances.

## References

[1] Nayak P.D.K. Two Days National Seminar on Alternative Energy Sources. Proceedings of V.P.M.’s Polytechnic.

[2] Steinfeld A. (2005). Solar thermochemical production of hydrogen–a review. Sol. Energy.

[3] Steinfeld A., Palumbo R., Meyers A. (2001). Solar Thermochemical Process Technology. Encyclopedia of Physical Science & Technology.

[4] Tritt T.M., Böttner H., Chen L. (2008). Thermoelectrics: Direct solar thermal energy conversion. Mater. Res. Bull..

[5] Yang J., Caillat T. (2006). Thermoelectric materials for space and automotive power generation. Mater. Res. Bull..

[6] Kim S.S., Yin F., Kagawa Y. (2006). Thermoelectricity for crystallographic anisotropy controlled Bi-Te based alloys and p-n modules. J. Alloys Compd..

[7] Yamashita O., Sugihara S. (2005). High-performance bismuth-telluride compounds with highly stable thermoelectric figure of merit. J. Mater. Sci..

[8] Reddy E.S., Noudem J.G., Hebert S., Goupil C. (2005). Fabrication and properties of four-leg oxide thermoelectric modules. J. Phys. D: Appl. Phys..

[9] Shin W., Muruyama N., Ikeda K., Sago S. (2001). Thermoelectric power generation using Li-doped NiO and (Ba, Sr)PbO_3_ module. J. Power Sources.

[10] Funahashi R., Mikami M., Mihara T., Urata S., Ando N. (2006). A portable thermoelectric-power-generating module composed of oxide devices. J. Appl. Phys..

[11] Funahashi R., Matsubara I., Ikuta H., Takeuchi T., Mizutani U., Sodeoka S. (2000). Oxide single crystal with high thermoelectric performance in air. Japan. J. Appl. Phys..

[12] Funahashi R., Urata S., Mizuno K., Kouuchi T., Mikami K. (2004). Ca_2.7_Bi_0.3_Co_4_O_9_/La _0.9_Bi_0.1_NiO_3_ thermoelectric devices with high output power density. Appl. Phys. Lett..

[13] Terasaki I., Sasago Y., Uchinokura K. (1997). Large thermoelectric power in NaCo_2_O_4_ single crystal. Phys. Rev. B.

[14] Ito M., Nagira T., Furumoto D., Katsuyama S., Nagai H. (2003). Synthesis of Na_x_Co_2_O_4_ thermoelectric oxides by the polymerized complex method. Scr. Mater..

[15] Zhou S., Zhao J., Chu S., Shi L. (2007). Synthesis, characterization and magnetic properties of lightly doped La_2-x_Sr_x_CuO_4_ (x = 0.04) nanoparticles. Phys. C.

[16] Bocher L., Robert R., Aguirre M.H., Malo S., Hébert S., Maignan A., Weidenkaff A. (2008). Thermoelectric and magnetic properties of perovskite-type manganate phases synthesised by ultrasonic spray combustion (USC). Solid State Sci..

[17] Weidenkaff A. (2004). Preparation and application of nanostructured perovskite phases. Adv. Eng. Mater..

[18] Aguirre M.H., Canulescu S., Robert R., Homazava N., Logvinovich D., Bocher L., Lippert T., Döbeli M., Weidenkaff A. (2008). Structure, microstructure, and high-temperature transport properties of La_1-x_Ca_x_MnO_3-δ_ thin films and polycrystalline bulk materials. J. Appl. Phys..

[19] Tomeš P., Robert R., Trottmann M., Bocher L., Aguirre M.H., Hejtmánek J., Weidenkaff A. (2010). Synthesis and characterization of new ceramic thermoelectrics implemented in a thermoelectric oxide module. J. Electron. Mater..

[20] Hirsch D., Zedtwitz P.V., Osinga T., Kinamore J., Steinfeld A. (2003). A new 75 kW high-flux solar simulator for high-temperature thermal and thermochemical research. J. Sol. Energy Eng..

[21] Bocher L., Aguirre M.H., Logvinovich D., Shkabko A., Robert R., Trottmann M., Weidenkaff A. (2008). CaMn_1-x_Nb_x_O_3_ (x ≤ 0.08) perovskite-type phases as promising new high-temperature n-type thermoelectric materials. Inorg. Chem..

[22] Snyder G.J. (2004). Application of the compatibility factor to the design of segmented and cascaded thermoelectric generators. Appl. Phys. Lett..

[23] Bramson M.A. (1968). Infrared Radiation—A Handbook of Applications.

[24] Rowe D.M., Min G. (1998). Evaluation of thermoelectric modules for power generation. J. Power Sources.

[25] Suter C., Tomeš P., Steinfeld A., Weidenkaff A. (2010). Heat transfer and geometrical analysis of thermoelectric converters driven by concentrated solar radiation. Materials.

[26] Lemonnier S, Goupil Ch., Noudem J., Guilmeau E. (2008). Four-leg Ca_0.95_Sm_0.05_MnO_3_ unileg thermoelectric device. J. Appl. Phys..

